# Construction of influenza virosome from influenza A H1N1 PR8

**DOI:** 10.1186/1753-6561-5-S1-P5

**Published:** 2011-01-10

**Authors:** Mehri Noori, Shirin Ghorbani, Abbas Jamali, Mohammad Shenagari, Hamidreza Hashemi, Masoumeh Tavassoti Kheiri

**Affiliations:** 1 Department of Biology, Alzahra University, Tehran, 1993891176, Iran; 2 Department of Virology (Influenza Unit), Pasteur Institute of Iran, Tehran, 1316943551, Iran; 3 Guilan University of Medical Science, Guilan, Iran

## 

Influenza is a major viral respiratory infection of humans, responsible for 300,000–500,000 annual deaths world-wide. The influenza viruses are competent of genetic variation, both by continuous, gradual mutation and by reassortment of genome segments between viruses. Antigenic drift is the gradual evolution of viral strains, due to frequent mutations of the surface glycoproteins hemagglutinin and neuraminidase. Novel and increasingly safer vaccines use well-characterized antigens. Conversely, these antigens are regularly too small to be highly immunogenic and would help from administration of a suitable adjuvant. The virosomes are reconstituted influenza virus envelopes devoid of central core and genetic in sequence. In this study we proposed to construct an influenza virosome structure from influenza A H1N1 PR8.

During the production process, MDCK cells were cultured then infected with influenza virus strain PR8 and finally the supernatant harvested and purified by ultracentrifugation and ultrafiltration. Purified influenza virus treated by DCPC as a detergent to resolve envelop of influenza virus. Then, RNP of influenza virus participated by using ultracentrifugation. The envelope of influenza virus was reconstituted by removing of DCPC by using overnight dialysis against HBS buffer. Finally, we observed empty influenza virus envelop by TEM, their called virosomes. The size of these particles was estimated between 50-150 nm.

